# Ilicicolin A Exerts Antitumor Effect in Castration-Resistant Prostate Cancer Via Suppressing EZH2 Signaling Pathway

**DOI:** 10.3389/fphar.2021.723729

**Published:** 2021-10-27

**Authors:** Lang Guo, Xiaowei Luo, Ping Yang, Yanting Zhang, Jialuo Huang, Hong Wang, Yinfeng Guo, Weifeng Huang, Zhiqiang Chen, Shusheng Wang, Junjian Wang, Jinping Lei, Songtao Xiang, Yonghong Liu

**Affiliations:** ^1^ Department of Urology, The Second Affiliated Hospital of Guangzhou University of Chinese Medicine, Second Clinical Medical College, Guangzhou University of Chinese Medicine, Guangzhou, China; ^2^ Institute of Marine Drugs/Guangxi Key Laboratory of Marine Drugs, Guangxi University of Chinese Medicine, Nanning, China; ^3^ Department of Pathology, Sun Yat-sen University Cancer Center, State Key Laboratory of Oncology in South China; Collaborative Innovation Center for Cancer Medicine, Guangzhou, China; ^4^ School of Pharmaceutical Sciences, Sun Yat-sen University, Guangzhou, China; ^5^ Guangdong Provincial Key Laboratory of New Drug Design and Evaluation, School of Pharmaceutical Sciences, Sun Yat-sen University, Guangzhou, China; ^6^ CAS Key Laboratory of Tropical Marine Bio-resources and Ecology/Guangdong Key Laboratory of Marine Materia Medica, South China Sea Institute of Oceanology, Chinese Academy of Sciences, Guangzhou, China

**Keywords:** ascochlorin derivatives, enhancer of zeste homolog 2, enzalutamide, castration-resistant prostate cancer (CRPC), androgen receptor

## Abstract

The Polycomb protein enhancer of zeste homolog 2 (EZH2) has critical roles in prostate cancer (PCa) progression and drug-resistance, which remains an obstacle for PCa treatment. Enzalutamide (ENZ) is a second-generation androgen receptor antagonist employed for treatment of metastatic castration-resistant prostate cancer A considerable proportion of tumors eventually develop resistance during treatment. Thus, agents that can overcome resistance to PCa are needed urgently. Ilicicolin A (Ili-A), an ascochlorin derivative isolated from the coral-derived fungus *Acremonium sclerotigenum* GXIMD 02501, shows antiproliferative activity in human PCa cells, but its mechanism of action against Castration-resistant prostate cancer is not known. Herein, RNA-sequencing showed the EZH2 pathway to be involved in PCa proliferation. Ili-A at low doses reduced the protein level of EZH2, leading to transcriptional change. Interestingly, Ili-A suppressed the binding of EZH2 to promoter regions in AR/serine/threonine polo-like kinase-1/aurora kinase A. Moreover, Ili-A could enhance the anticancer activity of enzalutamide in CRPC cancer models. These data suggest that Ili-A could be used in combination with enzalutamide to treat CRPC.

## Introduction

*Cancer* of the prostate gland, known commonly as “prostate cancer” (PCa), is the most commonly diagnosed cancer in men living in the United States PCa is responsible for ∼10% of all cancer-related deaths in men (Siegel et al., 2021). In China, PCa is the sixth most commonly diagnosed malignancy among men, it accounts for 3.35% of malignant tumors, and represents 2.1% of the total cancer-related mortality in men (Chen et al., 2016). During the past decade, surgery or androgen-deprivation therapy has become the first-line treatment for PCa patients (Litwin and Tan, 2017).

However, a proportion of patients fail to respond to medical castration initially, and nearly all patients who undergo androgen-deprivation therapy will progress eventually to metastatic castration-resistant prostate cancer (mCRPC). Taxane-based chemotherapies, next-generation antiandrogens, and biosynthesis inhibitors, such as enzalutamide (also named MDV3100), abiraterone acetate, and cabazitaxel, have been administered to patients who developed mCRPC (Litwin and Tan, 2017; Oudard et al., 2017; Thakur et al., 2018).

Enzalutamide is an androgen receptor (AR) antagonist that blocks several key steps in the AR signaling pathway: androgen binding to the AR, nuclear translocation of activated AR, and binding of activated AR with DNA (Tran et al., 2009; Baciarello and Sternberg, 2016; Scott, 2018). Despite the clinical efficacy of these drugs in mCRPC patients, the almost inevitable emergence of drug resistance hampers a definitive cure, possibly due to amplification or gain-of-function somatic mutations of AR, aberrant posttranslational modification of the AR protein, alternative splicing events that result in hyperactive receptors, and cofactor dysregulation and/or intracrine androgen synthesis (Watson et al., 2015). Considerable effort has been made on improving these weaknesses, but an efficient and efficacious method is lacking.

Microbial natural products and their derivatives are important sources for drug discovery (Choi et al., 2018). Most antibiotics in clinical practice are unmodified or modified derivatives of natural products produced by microorganisms to kill competing bacteria, and have been isolated originally from environmental sources (Liu et al., 2020). Therefore, it may be possible to search for “natural” anti-tumor components. Intriguingly, ilicicolin A (Ili-A) is an ascochlorin derivative isolated from theBeibu Gulf coral-derived fungus *Acremonium sclerotigenum* GXIMD 02501. Ili-A has been reported to be a promising anti-tumor drug in recent studies (Nirma et al., 2015; Sorres et al., 2018).

Aberrant methylation of DNA and histone modification are hallmarks of several cancer types. It has become evident that genes encoding epigenetic modifiers play a crucial role in normal development and cancer progression, and inhibitors of DNA methyltransferases, histone deacetylases thus methyltransferases are widely used in cancer therapy (Kovač et al., 2018). One type of histone methyltransferase, enhancer of zeste homolog 2 (EZH2), is an enzymatic catalytic subunit of polycomb-repressive complex 2 (PRC2), which catalyzes trimethylation of histone H3 at lysine 27 (H3K27me3) through its core components EZH1/EZH2, EED and SUZ12, and silences its target genes. EZH2 is overexpressed in many cancer types, such as cancer of the endometrium (Roh et al., 2020; Smith et al., 2019), and prostate gland (Varambally et al., 2008; Eich et al., 2020). AR, an important therapeutic target in PCa, remain highly expressed even when the tumor progressed to advanced stage. Previous study showed that EZH2 interacts directly with AR and activates them, which leads to regulation of AR expression and downstream targets (Liu et al., 2019). Inhibition of EZH2 expression enhances the efficacy of enzalutamide in enzalutamide-resistant PCa cells (Bai et al., 2019). Previous study identified the role of EZH2 in aggressive PCa, and suggested that EZH2 serves as a target of PCa treatment (Sellers and Loda, 2002). Here, we found that Ili-A inhibited the transactivation activity of AR and their downstream targets. A low dose of Ili-A inhibited the growth of CRPC cells and enhance the anticancer activity of enzalutamide in CRPC cancer models. Thus, combination of Ili-A with enzalutamide could be add in treatment strategy for CRPC.

## Materials and Methods

### Cell Culture and Chemicals

22Rv1 was from American Type Culture Collection (ATCC, Manassas, VA, United States). C4-2B was from UroCor Inc. (Oklahoma City, OK, United States). C4-2B and 22Rv1 cells were cultured in RPMI 1640 medium (Senrui, Zhejiang, China). All culture media were supplemented with 10% fetal bovine serum and 1% penicillin/streptomycin (Gibco, Grand Island, NY, United States). Cells were cultured at 37°C in a humidified incubator containing 5% CO_2_. Ilicicolin A (Ili A) was isolated from the Beibu Gulf coral-derived fungus *Acremonium sclerotigenum* GXIMD 02501. The strain GXIMD 02501 was fermented on a nutrient-limited culture medium (10 g soluble starch, 1 g bacterial peptone, 20 g sea salt, and 1 L H2O) inoculating at room temperature with 1 L × 160 Erlenmeyer flasks. In brief, ilicicolin A was isolated from its EtOAc extract (20 g) by various chromatographic methods, including silica gel, reversed-phase silica gel C18, and semi-preparative HPLC. It was determined to have ≥95% purity by analytical HPLC. The chemical structure of ili A was further determined by comparing the spectroscopic data (Supplementary Figure S3) with the literature (Sorres et al., 2018). Other chemicals were purchased from Sigma Aldrich (St. Louis, MO, United States) unless specified otherwise.

### Cell Viability

Cells were seeded in 96-well plates at 500–1,000 cells per well (optimum density for growth) in a total volume of 100 *μ*L of media. Serially diluted compounds in 250 *μ*L of media were added 50 μL to the cells per well 24 h later. After 4 days of incubation, Cell-Titer GLO reagents (Promega Corp., Madison, WI, United States) were added, and luminescence was measured on GLOMAX microplate luminometer (Promega Corp.) according to the manufacturer’s instructions. The results were presented as percentages and vehicle-treated cells set at 100. The estimated *in vitro* IC_50_ values were calculated using GraphPad Prism seven software.

### Colony Formation

For colony-formation assays, 500 cells were seeded in a six-well plate and cultured for 10–14 days in a 37°C incubator; the medium was changed and the test compound added every 3 days. Cells were fixed in 4% paraformaldehyde for 15 min. Then, the plate was washed thrice with phosphate-buffered saline (PBS). Cell colonies were stained with crystal violet for 15 min. The number of colonies was counted after washing thrice with PBS. Colony-formation assays were carried out in triplicate, and all experiments were repeated three times.

### RT-qPolymerase Chain Reaction

Total RNA was extracted from cells in 6-well plates. cDNA was prepared according to the manufacturer’s protocol. PCR was performed on a BIO-RAD CFX96™ (BIO-RAD, San Diego, CA, United States) in the presence of SYBR Green. After the fluorescence values were collected, a melting-curve analysis was performed. The experiments were performed with data presented as mean standard deviation (SD). The sequences of primers for the RT-qPCR analysis are listed in Supplementary Table S1.

### Cell Lysates and Western Blot Analysis

The 22Rv1, C4-2B, and C4-2B/ENZR cells were treated with the vehicle or different concentrations of ilicicolin A (Ili-A) or ENZ for 48 h before being harvested for a Western blot analysis. The cells were lysed in RIPA buffer plus 1% PMSF (Phenylmethylsulfonyl fluoride) and then were separated on a denaturing polyacrylamide gel according to the manufacturer’s instructions. The proteins were transferred to a PVDF (Polyvinylidene fluoride) membrane and were blocked for 1 h in a 5% nonfat milk solution (Trisbuffered saline, 0.1% Tween (TBS-T), 5% nonfat dry milk). Finally, the signal was detected using a BIO-RAD imager. All the antibodies used in this study are described in the Supplementary Table S2.

### Chromatin Immunoprecipitation Assay

Crude chromatin solutions of C4-2B cells were cleared with protein-A beads (Invitrogen, Carlsbad, CA, United States) that had been precoated with preimmune serum (i.e., serum extracted before immunization) for 2 h at 4°C. Then, the precleared chromatin solutions were incubated overnight at 4°C with antibodies (listed in Supplemental Material) before precipitation with protein-A beads that had been preblocked with bovine serum albumin and sonicated salmon-sperm DNA. For reChIP, the immunoprecipitated complexes from the first ChIP were eluted with dithiothreitol (20 mM) for 30 min at 37°C with brief vortex-mixing, diluted 50 times with ChIP buffer, cleared by centrifugation, and incubated with antibodies for secondary ChIP overnight at 4°C. The immunoprecipitated DNA was analyzed by real-time reverse transcription-quantitative polymerase chain reaction (RT-qPCR) with SYBR^®^ Green on an iCycler^®^ (Thermo Fisher, Waltham, MA, United States). Enrichment of genomic DNA is presented as the percent recovery relative to the input. The primers are listed in Supplementary Table S1.

### EZH2 Activity Assessed Step

Dilute EZH2 enzyme, SAM(S-(5′-Adenosyl)-l-methionine chloride), compounds and peptide substrate in Assay Buffer just before use. Add 4 *μ*L of inhibitor, 2 *μ*L of enzyme to the wells of a white OptiPlate-384 and incubate for 10 min at room temperature. Subsequently, add 4 *μ*L of H3 (1–50) K27 me0-biotin peptide/SAM mix to the reaction system. Cover the plate with TopSeal-A film and incubate at room temperature for 4 h. Prepare a 2X mix of anti-H3K27me3-Eu(K) at 0.225 μg/ml and SA-XL 665 at 10 ng/*μ*L, respectively, in Detection Buffer. Add 10 *μ*L of detection mixture (2X) to the plate. Cover with TopSeal-A film and incubate in subdued light for 1 h at room temperature. Remove plate sealer and read fluorescence emission at 665 and 620 nm wavelengths on an HTRF compatible reader. The resultant data were analyzed with GraphPad Prism.

### Molecular Docking

The crystal structure of human PRC2 (Protein Data Bank (PDB) code: 5WG6), which includes the EZH2 subunit, was downloaded from PDB (www.pdb.org/) and used for molecular docking. Preparation of the protein structure (protonation-state adjustment, water deletion, addition of disulfide bonds and hydrogen atoms) was undertaken using Maestro 11.6.013 (Schrodinger, New York, NY, United States). The ligand was also prepared by Maestro, and the “Glide” docking program in Maestro was used for docking studies. The designed molecule Ili-A was first docked into the catalytic region of EZH2 using Glide SP mode. Then, the predicted binding mode was redocked further into this region by Glide XP mode. For docking and redocking using Glide, the grid was defined using a 30-A box centered on the N atom of residue TRP624 in the catalytic site of EZH2. All other parameters were kept as default. PyMOL (DeLano Scientific, Palo Alto, CA, United States) was used to obtain the three-dimensional structure of the docking model.

### Ribonucleic Acid Sequencing Data Analysis

C4-2B cells were treated with vehicle or the small molecule Ili-A (5, 10 *μ*M), for 48 h before RNA extraction. RNA-sequencing libraries were from 1 *μ*g of total RNA, which was extracted using the Total RNA Isolation kit from Tiangen (Beijing, China) and reverse-transcribed with reverse transcriptase M-MLV according to manufacturer (TaKaRa Biotechnology, Shiga, Japan) instructions. Libraries were validated with a bioanalyzer (2,100 series; Agilent Technologies, Palo Alto, CA, United States). Sequencing was done on a MGISEQ 2000 SE 50 system (BGI Tech, Wuhan, China). First, we used Trim Galore (www.bioinformatics.babraham.ac.uk/)! to automate quality and adapter trimming, as well as quality control, for FastQ files. Then, the FastQ-formatted sequence data were analyzed using a standard BWA-Bowtie 2-Cufflinks workflow. Briefly, sequence reads were mapped to the reference human-genome assembly (GRCh37/hg19) with BWA and Bowtie 2. Subsequently, the Cufflinks package 62 was applied for transcript assembly, quantification of normalized gene and isoform expression in fragments per kilobase of exon model per million mapped reads (FPKM), and testing for differential expression (Cuff diff). To avoid spurious fold levels resulting from low expression values, only those genes with expression FPKM >1 for the vehicle control cell or treated cells (but not necessarily both), were included. Change in expression of ≥1.5-fold (increase or decrease) was clustered with the k-means clustering algorithm in Cluster 63. The cluster was displayed with Tree View.

### Animal Experiments

For establishing the prostate cancer xenograft tumors, Four-week-old male NOD/SCID mice (body weight 18 g) were purchased from Zhongshan Biomedical Research Institute of Sun Yat-Sen University (Zhongshan, China). Briefly, the mice were injected with 10× 10^7^ 22Rv1 cells in 100 *μ*L PBS/Matrigel (1:1), and implanted subcutaneously into the dorsal flank of the mice. When the tumor volume was reached about 50 mm^3^, the mice were grouped randomly. Then mice were divided into four groups (*n* = 8) randomly and treated intraperitoneally (i.p.) with 100 *μ*L of either vehicle (DMSO and sesame oil, 1:50, Sigma, St. Louis, MO, United States), 10 mg/kg Ilicicolin A (Ili-A) (six times a week), 10 mg/kg Enzalutamide (six times a week) or a combination of 10 mg/kg Ilicicolin A (Ili-A) (six times a week) and 10 mg/kg Enzalutamide (six times a week). Tumor volume and body weight were measured three times per week. Tumor growth was monitored by calipers with volume calculated using the equation π/6 (length × width^2^). The mice were sacrificed at the end of the studies. Tumors were collected and immediately stored in liquid nitrogen or fixed in formalin solution. The procedures were approved by the Institutional Animal Care and Use Committee of Sun Yat-Sen University (Guangzhou, China).

### Animal Experiments

For establishing the prostate cancer xenograft tumors, Four-week-old male NOD/SCID mice (body weight 18 g) were purchased from Zhongshan Biomedical Research Institute of Sun Yat-Sen University (Zhongshan, China). Briefly, the mice were injected with 10× 10^7^ 22Rv1 cells in 100 *μ*L PBS/Matrigel (1:1), and implanted subcutaneously into the dorsal flank of the mice. When the tumor volume was reached about 50 mm^3^, the mice were grouped randomly. Then mice were divided into four groups (*n* = 8) randomly and treated intraperitoneally (i.p.) with 100 *μ*L of either vehicle (DMSO and sesame oil, 1:50, Sigma, St. Louis, MO, United States), 10 mg/kg Ilicicolin A (Ili-A) (six times a week), 10 mg/kg Enzalutamide (six times a week) or a combination of 10 mg/kg Ilicicolin A (Ili-A) (six times a week) and 10 mg/kg Enzalutamide (six times a week). Tumor volume and body weight were measured three times per week. Tumor growth was monitored by calipers with volume calculated using the equation π/6 (length × width^2^). The mice were sacrificed at the end of the studies. Tumors were collected and immediately stored in liquid nitrogen or fixed in formalin solution. The procedures were approved by the Institutional Animal Care and Use Committee of Sun Yat-Sen University (Guangzhou, China).

### Statistical Analyses

Analyses were conducted with Prism 7 (GraphPad, San Diego, CA, United States). Differences between two groups were analyzed by Student’s t-test. *p* < 0.05 was considered significant. The results are expressed as the mean ± standard deviation (SD).

## Results

### Ili-A Inhibits the Survival of Castration-Resistant Prostate Cancer Cells and Induces Their Apoptosis

**FIGURE 1:**
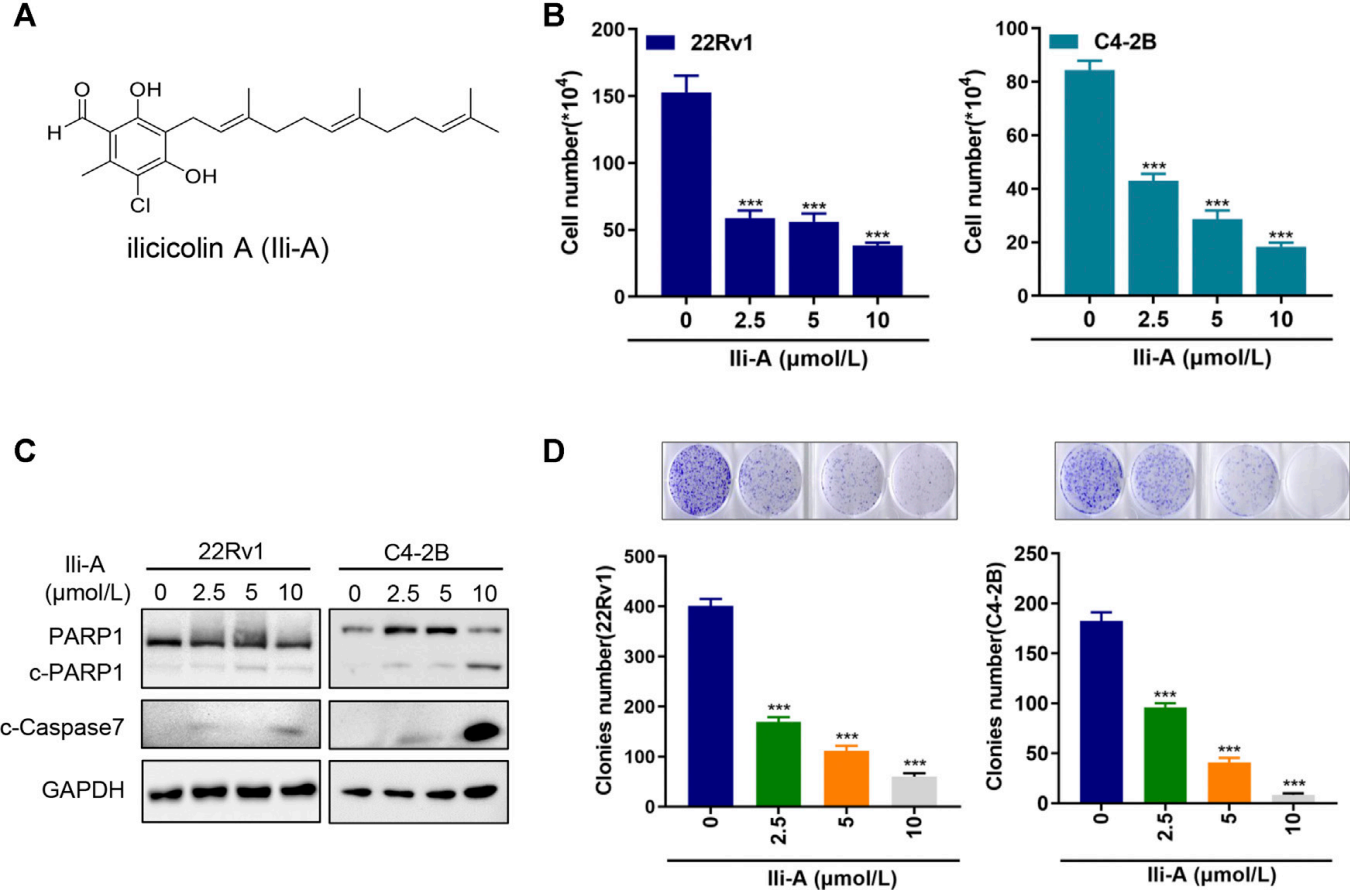
Ili-A inhibits survival of castration-resistant prostate cancer cells and induces their apoptosis. **(A)** Chemical structure of Ili-A. **(B)** 22Rv1 cells and C4-2B cells were treated with vehicle or Ili-A as indicated. After 96 h, the total number of viable cells was counted with a cell counter. **(C)** Immunoblotting of the indicated proteins in 22Rv1 and C4-2B cells treated with vehicle or Ili-A for 48 h. **(D)** 22Rv1 cells and C4-2B cells were treated with vehicle (DMSO) or the indicated concentrations of Ili-A for 12 days, after which colony formation was counted.

We wished to search for drug candidates for CRPC treatment in humans. We screened a chemical library containing 30 natural compounds from marine microorganisms for their activity in growth inhibition of CRPC cell lines (C4-2B and 22Rv1) (Supplementary Figures S1A,B). Chemical structure of Ili-A (Figure 1A) and showed a strong chemotherapeutic effect in C4-2B cells and 22Rv1 cells at nanomolar concentrations (Figure 1B). Ili-A could induce expression of apoptosis-related proteins such as cleaved- Poly [ADP-ribose] polymerase (PARP)-1 and cleaved-caspase 7 (Figure 1C). A similar result was observed for formation of cell colonies (Figure 1D).

### Ili-A Inhibits Expression of a EZH2-Mediated Signaling Pathway

**FIGURE 2:**
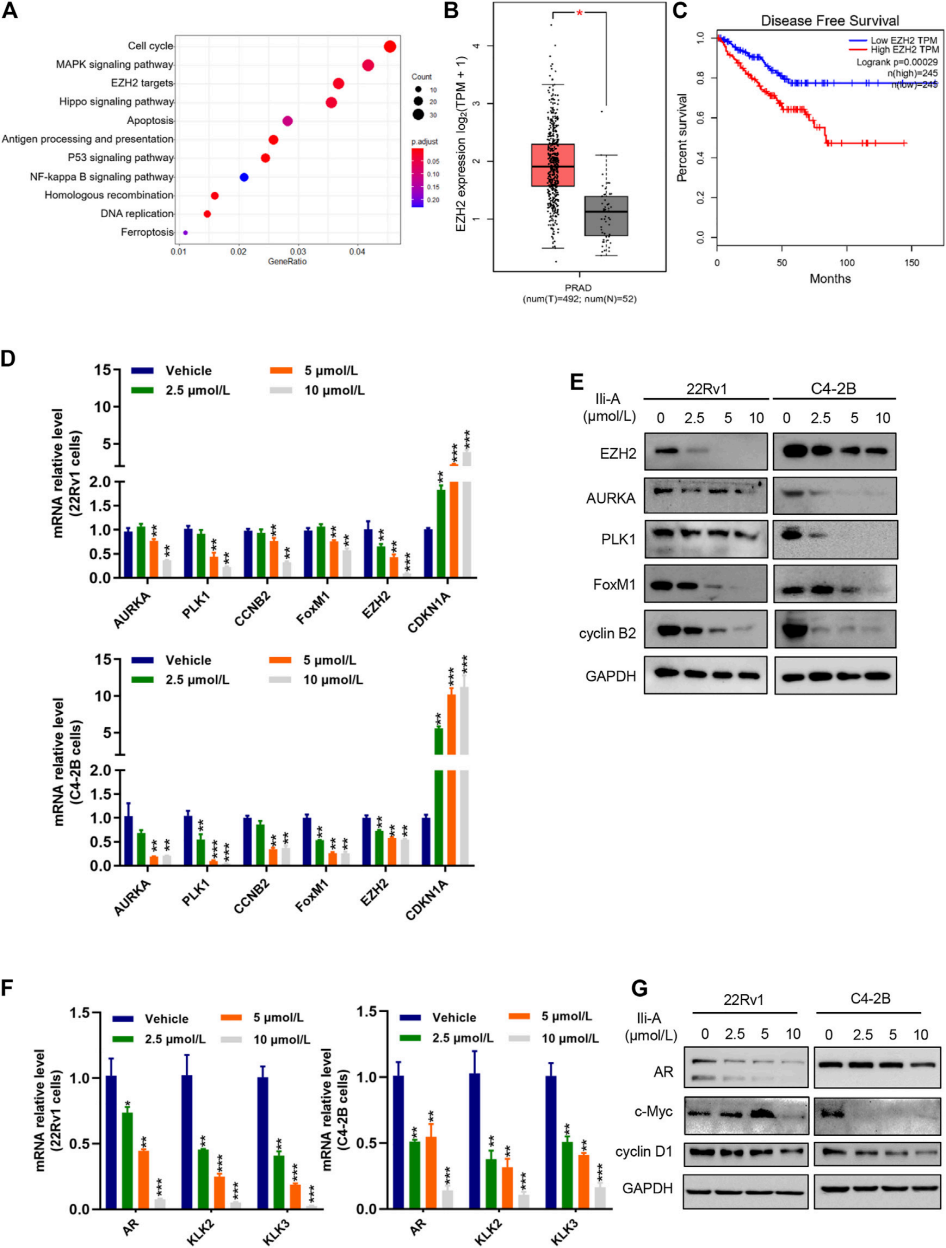
RNA-sequencing shows that the EZH2 pathway is involved in the proliferation of prostate cancer cells. **(A)** Bubble chart showing enrichment of the most significantly changed genes in the signaling pathways of C4-2B cells. **(B)** Gene expression of EZH2 expression in human prostate cancer in TCGA and matched normal prostate-gland tissues in TCGA using log2 (TPM +1) for log scale. **(C)** EZH2 mRNA expression was correlated inversely with survival from prostate cancer. Kaplan–Meier plots are based on analyses of data from prostate-cancer patients in TCGA using GEPIA. **(D)** RT-qPCR showing expression of the indicated cyclin genes in 22Rv1 cells and C4-2B cells treated with vehicle or Ili-A for 48 h. **(E)** Immunoblotting showing expression of the indicated cyclin proteins in 22Rv1 cells and C4-2B cells treated with vehicle or Ili-A for 48 h. **(F)** RT-qPCR showing expression of the AR downstream genes in 22Rv1 cells and C4-2B cells treated with vehicle or Ili-A for 48 h. **(G)** Immunoblotting showing expression of AR downstream proteins in 22Rv1 cells and C4-2B cells treated with vehicle or Ili-A for 48 h.

We added Ili-A to C4-2B cells and extracted RNA. The amplified PCR product was sent for sequencing. Using the Kyoto Encyclopedia of Genes and Genomes (KEGG) database, we discovered that 11 pathways were enriched significantly among PCa genes. Of these, EZH2-targeting pathways were mainly enriched (Figure 2A). Based on the *Cancer* Genome Atlas database, EZH2 was overexpressed in PCa (Figure 2B) and associated significantly with an unfavorable prognosis of patients (Figure 2C). EZH2 has a pivotal role in the development and progression of PCa, so next we examined whether Ili-A could inhibit survival of CRPC cells by blocking EZH2 signaling. Ili-A significantly inhibited expression of EZH2 at mRNA and protein levels in a dose-dependent manner (Figures 2D,E). Ili-A deceased expression of proteins associated with cellular proliferation and the cell cycle, such as cyclin B2 and serine/threonine polo-like kinase (PLK)1. To investigate AR function by EZH2, we examined if Ili-A could repress AR transcription by blocking EZH2 signaling. Ili-A significantly inhibited expression of AR signaling at mRNA and protein levels in a dose-dependent manner (Figures 2F,G).

### Ili-A Degrades EZH2 Protein by Promoting the Ubiquitin–Proteasome Pathway

**FIGURE 3:**
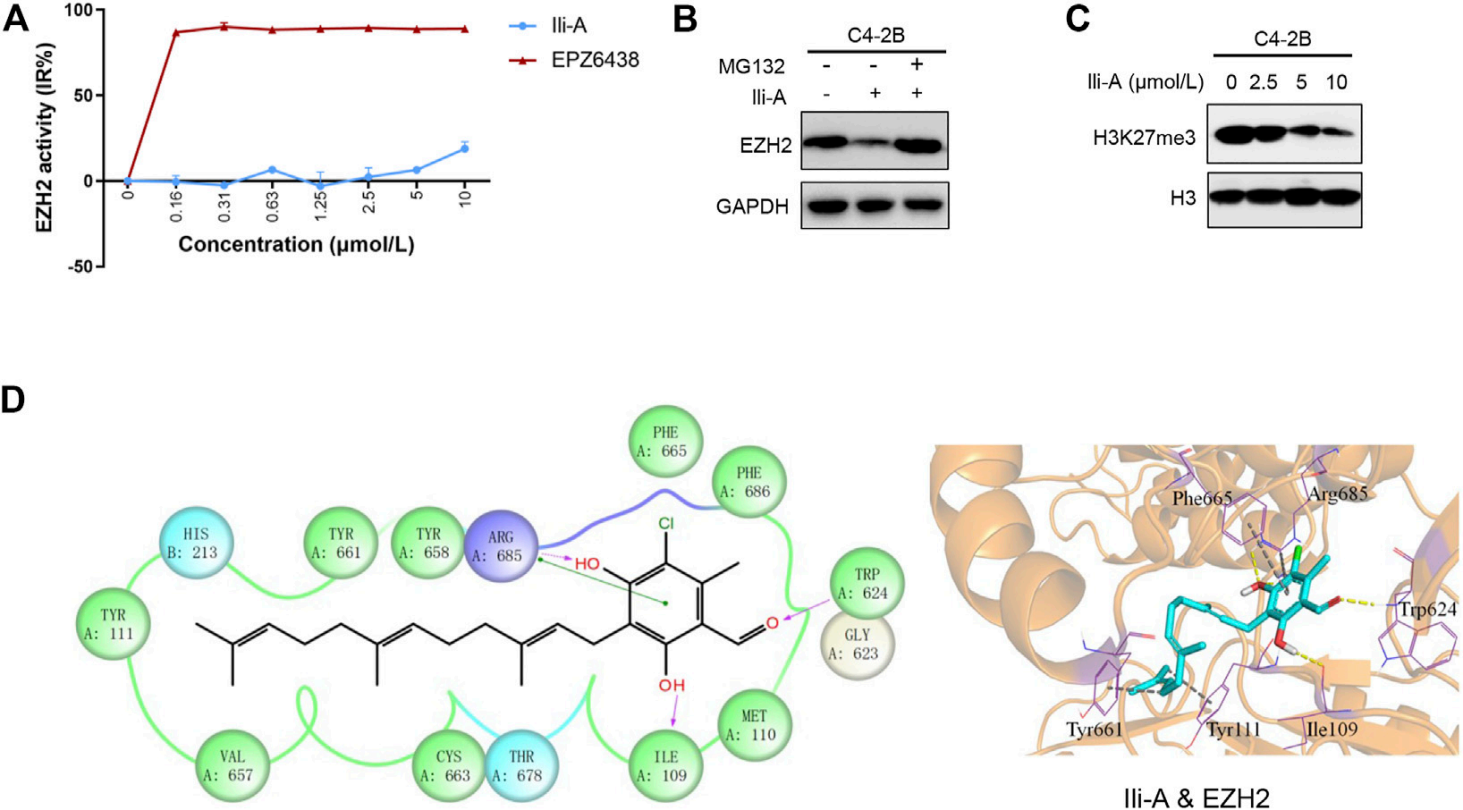
Ili-A inhibits expression of EZH2-mediated signaling. **(A)** Biochemical inhibition of full-length human EZH2 by compound Ili-A and EPZ6438. **(B)** Immunoblotting showing an EZH2 protein-degraded pathway in C4-2B cells treated with vehicle or Ili-A for 18 h and MG132 for an additional 6 h. **(C)** Immunoblotting showing bulk histone methylation for H3K27me3 on acid-extracted histones in C4-2B cells treated with vehicle or Ili-A for 48 h. **(D)** Binding mode of Ili-A in the catalytic region of EZH2 by molecular docking. The hydrogen bonds are labeled by yellow dashed lines, and the hydrophobic interaction is shown as gray dashed lines.

We investigated the functional importance of EZH2 expression in the EZH2 signaling pathway by characterizing the inhibition effect of Ili-A on EZH2 activity. Surprisingly, EZH2 activity was uninhibited even after administration of Ili-A in nanomolar concentrations (Figure 3A). However, protein expression of EZH2 was decreased according to western blotting, by conduct MG132 (proteasome inhibitor), EZH2 protein expression was recovered. Hence, Ili-A may mediate EZH2 signaling by degrading EZH2 protein and promoting the ubiquitin–proteasome pathway (Figure 3B).

EZH2 is an important catalytic subunit of the PRC2 complex. It contains a SET catalytic domain, which is a methyltransferase domain that functions as exogenous methyl group identification through covalent reaction in combination of catalytic histone H3 lysine 9 and 27 occurred three methylation (H3K9me3 and H3K27me3). EZH2 is a key tumor-suppressor gene that induces chromatin densification and epigenetic silence, then leads to tumorigenesis. Thus, we also examined the trimethylation of H3K27me3. We discovered that inhibition of EZH2 expression with Ili-A decreased H3K27me3 expression in C4-2B cells (Figure 3C). To further understand the inhibition mechanism of human EZH2 with Ili-A at the molecular level, we undertook molecular docking to investigate the detailed interactions between Ili-A and EZH2. Ili-A was docked into the split catalytic region of EZH2 comprising the SET domain (residues 623–625 and 657–686) and SET activation loop (SAL; residues 109–111) (Figure 3D) (Bratkowski et al., 2018). Ili-A could bind tightly to the catalytic region of EZH2 in a similar manner to that seen with pyridone inhibitors (Figure 3D). The carbonyl oxygen of the phenyl head of Ili-A formed a hydrogen bond with main-chain amide of Trp624, so Ili-A could compete with S-adenosylmethionine (SAM) binding to the same residue (Trp624) of EZH2. In addition, the two hydroxyl groups in the phenyl head of Ili-A could form a hydrogen bond with Arg685 in the drug-binding pocket and Ile109 in the SAL region, respectively. Furthermore, the alkane tail of Ili-A extended to the SET/SAL gate comprising Tyr661 and Tyr111, and could form strong hydrophobic interactions with the benzene ring of these two residues. Therefore, our molecular-docking results indicated that Ili-A could act as a novel EZH2 inhibitor and compete with SAM for EZH2 binding as general pyridone-containing inhibitors.

### Ili-A Decreases Expression of EZH2 Target Genes via the EZH2 Signaling Pathway

**FIGURE 4:**
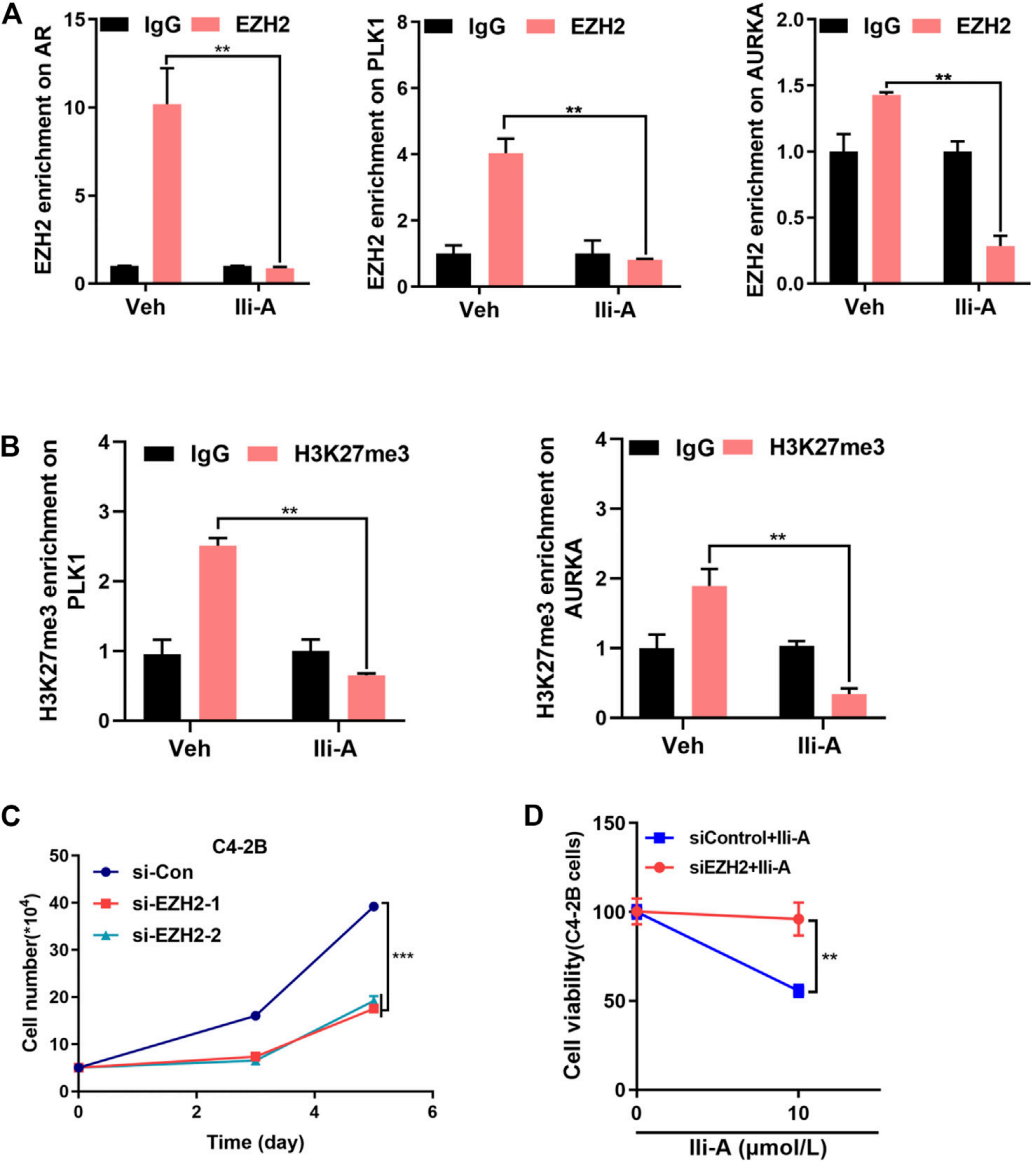
Ili-A decreases expression of EZH2 target genes by inhibiting EZH2 expression. **(A)** ChIP-qPCR of the relative enrichment of EZH2 at *AR*, *PLK1* and *AURKA* promoters in C4-2B cells treated with vehicle or Ili-A for 48 h **(bottom)**. “Fold change” denotes the indicated enrichment of *AR*, *PLK1*, and *AURKA* under the influence of Ili-A compared with IgG enrichment in cells treated with vehicle control set as 1. **(B)** ChIP-qPCR of the relative enrichment of H3K27me3 at *PLK1* and *AURKA* promoters in C4-2B cells treated with vehicle or Ili-A for 48 h **(bottom)**. Fold change denotes the indicated enrichment on *PLK1* and *AURKA* under the influence of Ili-A compared with IgG enrichment in cells treated with vehicle set as control. **(C)** C4-2B cells were transfected with EZH2 or control siRNA for 72 and 120 h, and the total number of viable cells was counted with a cell counter. **(D)** C4-2B cells were transfected with EZH2 or control siRNA for 48 h and treated with vehicle or Ili-A for an additional 24 h. Cells were harvested to determine cell growth by counting the number of viable cells.

ChIP-qPCR showed that Ili-A strongly reduced EZH2 occupancy on *AR*/*PLK1*/*AURKA* promoter sites (Figure 4A). Given that EZH2 causes methylation of H3K27me3, our data showed that Ili-A reduced H3K27me3 occupancy on *PLK1*/*AURKA* promoter sites (Figure 4B).

We wished to ascertain if silencing of EZH2 expression would block inhibition of Ili-A and affect cell survival. First, we used EZH2 small interfering (si)RNA to silence EZH2 expression specifically. Ili-A-induced inhibition of cell survival was attenuated significantly in EZH2 siRNA-treated cells compared with that in control cells (Figures 4C,D). Collectively, these results suggested that Ili-A could inhibit proliferation of CRPC cells by inhibiting EZH2 expression-mediated target-gene expression.

### Ili-A Sensitizes Castration-Resistant Prostate Cancer Cells to Enzalutamide Induced Cell Death *In Vitro*

**FIGURE 5:**
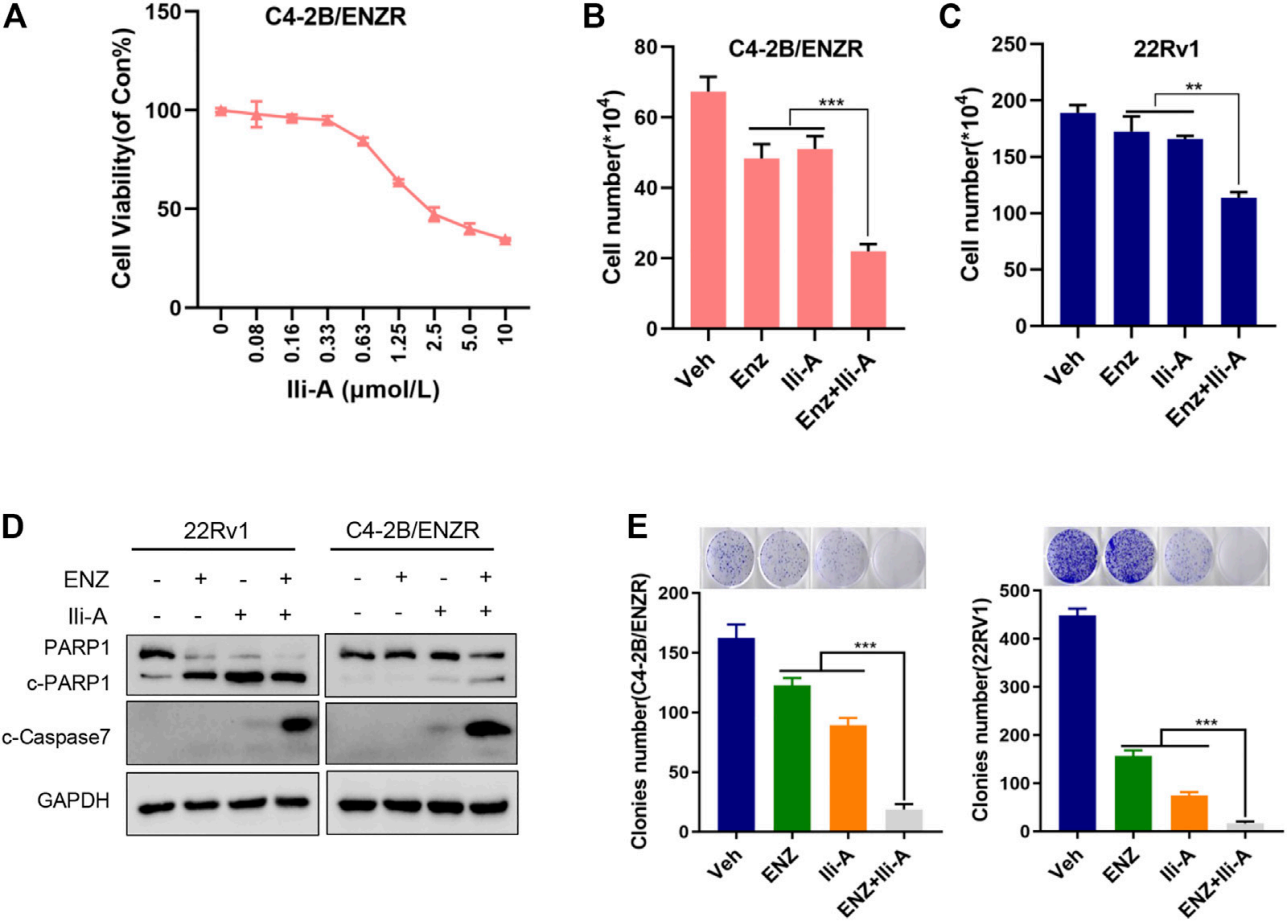
Cotreatment of Ili-A and enzalutamide (ENZ) suppresses cell growth and induces apoptosis in CRPC cells. **(A)** Cell proliferation was assessed using the CellTiter-Glo assay after 96 h of treatment with Ili-A in C4-2B/ENZR cells at the indicated doses. **(B,C)** Cell numbers were counted with a cell counter after 96 h of treatment with Ili-A (1.25 *μ*M), enzalutamide (ENZ; 10 *μ*M) or Ili-A (1.25 *μ*M) plus ENZ (10 *μ*M) in 22Rv1 cells and C4-2B/ENZR cells. **(D)** Western blots demonstrating apoptotic markers following treatment with vehicle, Ili-A (1.25 *μ*M), enzalutamide (ENZ; 10 *μ*M) or Ili-A (1.25 *μ*M) plus ENZ (10 *μ*M) in 22Rv1 cells and C4-2B/ENZR cells. **(E)** Cell proliferation as assessed using a colony-formation assay after 12 days of treatment with Ili-A (1.25 *μ*M), enzalutamide (ENZ; 10 *μ*M) or Ili-A (1.25 *μ*M) plus ENZ (10 *μ*M) in 22Rv1 cells and C4-2B/ENZR cells.

To further explore the effects of Ili-A on CRPC cells, we generated an enzalutamide-resistant PCa cell line C4-2B/ENZR (C4-2 enzalutamide-resistant) by continuous culture of C4-2 cells in a medium containing enzalutamide for 7 months. C4-2B/ENZR cells showed more resistance to enzalutamide than the parental C4-2 cells. Interestingly, we found that the viability of C4-2B/ENZR cells was reduced significantly by Ili-A treatment in a dose-dependent manner (Figure 5A). This observation indicated that Ili-A: 1) could overcome the enzalutamide-induced resistance of PCa cells; 2) could combine with enzalutamide against CRPC. To support this hypothesis and explore the combined effects of these two agents, we treated 22Rv1 cells with Ili-A (1.25 *μ*M) in the presence or absence of enzalutamide (10 *μ*M) for 4 days. Combination of Ili-A and enzalutamide had a stronger inhibitory effect on the viability of 22Rv1 cells than that observed with treatment of Ili-A alone or enzalutamide alone (Figure 5B). Similar results were observed in the enzalutamide-resistant cell line C4-2B/ENZR (Figure 5C). Interestingly, co-treatment also induced higher expression of the cleaved products of two well-known markers of apoptosis, cleaved-PARP-1 and cleaved-caspase 7, in 22Rv1 cells and C4-2B/ENZR cells (Figure 5D), indicating that such combined treatment might be more toxic to CRPC cells than monotherapy. Moreover, co-treatment with Ili-A and enzalutamide had a stronger inhibitory effect on the colony-forming ability of 22Rv1 cells and C4-2B/ENZR cells (Figure 5E). Taken together, these results indicated that Ili-A may have a synergistic effect with enzalutamide against PCa cells *in vitro*.

### Ili-A Inhibits Growth of Castration-Resistant Prostate Cancer Tumors and Potentiates the Anti-Tumor Effect of Enzalutamide *In Vivo*

**FIGURE 6:**
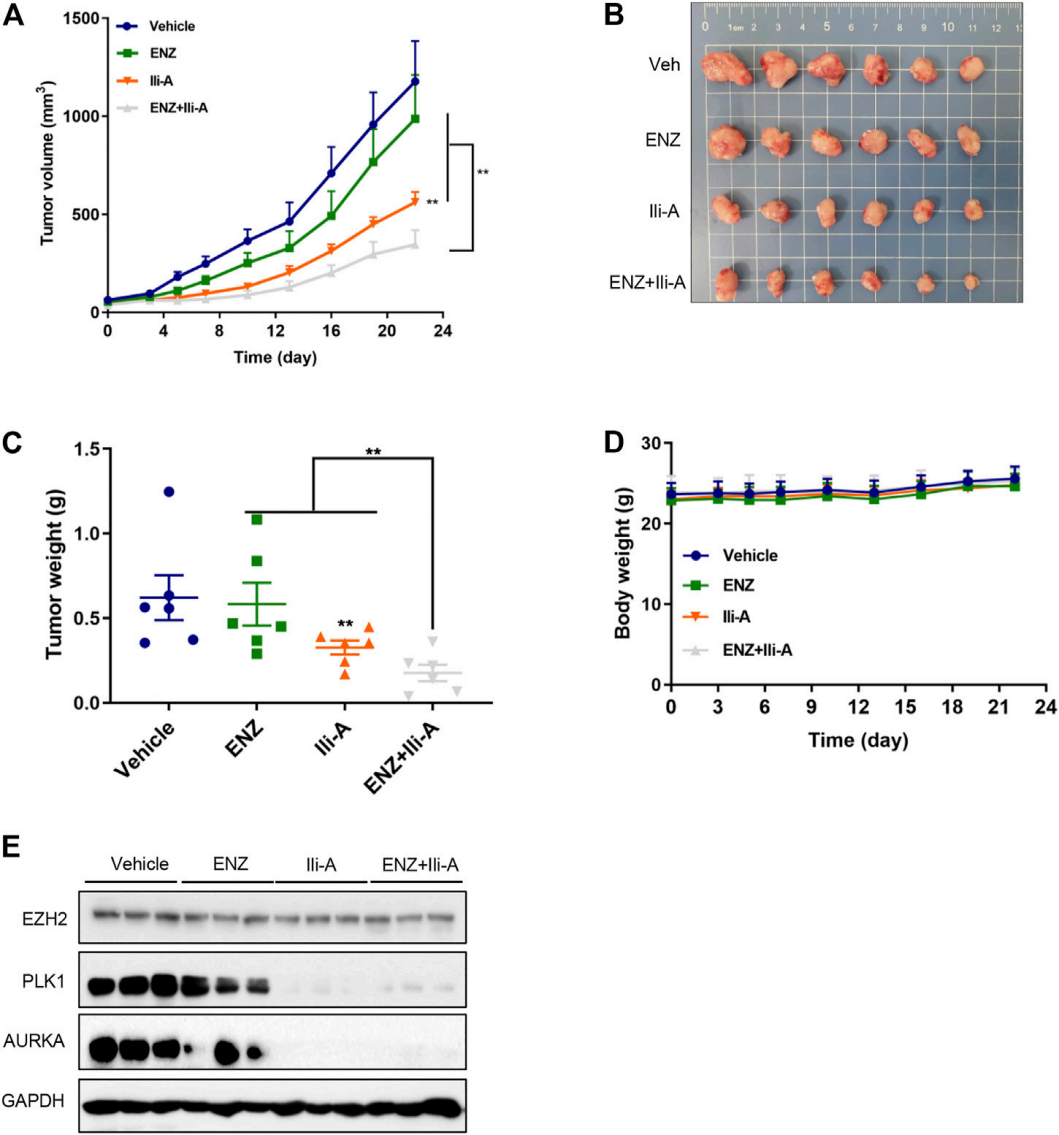
Ili-A enhances the anti-tumor effects of enzalutamide *in vivo*. Mice bearing 22Rv1 xenografts (*n* = 8 mice per group) received vehicle, Ili-A (10 mg/kg, i. p.), ENZ (10 mg/kg, i. p.) or a combination of Ili-A and ENZ, as indicated, six times per week. **(A)** Tumor volume in different groups (tumor volume was measured twice every week). **(B)** Images of xenograft tumors harvested at day-22. **(C)** Weight of xenograft tumors in different groups. **(D)** Body weight of Mice in different groups. **(E)** Western blotting was done to detect EZH2 and target proteins in tumors undergoing monotherapy or combination with Ili-A and enzalutamide.

To investigate the anti-tumor effects of co-treatment of Ili-A and enzalutamide *in vivo*, 22Rv1 cells were used to establish an animal xenograft model of CRPC. Male nude mice bearing 22Rv1 xenografts were randomized and treated with Ili-A (10 mg/kg; six times per week), enzalutamide (10 mg/kg; six times per week), or both, for 3 weeks. Tumor volume and bodyweight were monitored twice or thrice a week. At the end of 3 weeks, mice were sacrificed and xenografts were harvested for further investigation. After 3 weeks of combined treatment with Ili-A and enzalutamide, the volumes and weight of xenograft tumors decreased dramatically compared with the moderate effect of Ili-A alone and the slight effect of enzalutamide alone (Figures 6A–C). Photographs of the heart, spleen, and kidneys showed no visible change after drug treatment (Supplementary Figure S2). The three treatment groups suppressed tumor growth to different extents but did not elicit a significant effect on bodyweight (Figure 6D). Moreover, *EZH2*/*PLK1*/*AURKA* expression was strongly suppressed in the co-treatment group (Figure 6E). These results indicated that Ili-A could enhance the anti-cancer effect of enzalutamide on CRPC cells *in vivo*.

## Discussion

Despite significant progress in anti-androgen therapies for CRPC, as a second-generation AR antagonist, enzalutamide has been widely used in treatment of advanced prostate cancer. However, the acquired resistance to these therapies is inevitable, which restricts treatment efficacy. Thus, the discovery of new therapeutic targets against CRPC resistance is an urgent and unmet need. In this study, we showed that EZH2 plays an important role in the development of PCa and enzalutamide resistance. We demonstrated that Ili-A could be a novel EZH2 antagonist that exerts anti-tumor effects against CRPC *in vitro* and *in vivo* and capable of enhance the anticancer activity of enzalutamide in CRPC cancer models. Mechanistically, we demonstrated that Ili-A exerts its effect by inhibiting EZH2 expression and AR activation. These findings were highly consistent in our prostate cancer cell models *in vitro* and 22Rv1 xenograft mice models *in vivo*, suggesting that combination of Ili-A and enzalutamide would be more efficient in treating patients with advanced prostate cancer, especially the ones with enzalutamide-resistant cancer.

Several naturally occurring antagonists, mainly derived from marine microorganisms, have shown potent anti-tumor activity. Chemical activities in growth inhibition of C4-2B cells and 22Rv1 cells show that Ili-A displayed chemotherapeutic effect in C4-2B cells and 22Rv1 cells at nanomolar concentrations. C4-2B cells were treated with Ili-A, and RNA-sequencing carried out. Use of the KEGG database revealed that EZH2-targeting pathways were the main ones enriched. Given its crucial roles in the development and progression of PCa, EZH2 could be a novel therapeutic target for CRPC.

It is well established that up-regulation of EZH2 is closely correlated with the progression of advanced PCa and unfavorable outcome, but the EZH2 inhibitors-based treatment is basically ineffective for PCa (Deb et al., 2013). EZH2 and other PRC2 components are transcriptional repressors that methylate H3K27 and condense chromatin conformation. Scholars have reported that EZH2 can repress multiple downstream targets (e.g., *KDM6A*, *GIT1*) directly by binding to their promoter regions contributed to the progression of urothelial bladder carcinoma (Ler et al., 2017; Yang et al., 2020). In CRPC cells, PLK1 is a regulator of several cell-cycle events: mitotic entry, formation of bipolar spindles, and cytokinesis (Strebhardt, 2010). *AURKA* is a serine/threonine protein kinase crucial for formation of mitotic spindles and chromosome segregation (Willems et al., 2018). By directly binding to the promoter region of *PLK1* and *AURKA*, EZH2 serves as a regulatory transcriptional factor in regulation of the expression of *PLK1* and *AURKA* and subsequently induce proliferation of PCa. In our study, we observed that Ili-A could reduce EZH2 protein and mRNA level and the expression of the downstream *AURKA*/PLK1 gene, which contribute to the anti-tumor effects against CRPC.

Compounds capable of degrading EZH2 protein (similar to knockdown of EZH2 expression) might greatly outperform enzymatic EZH2 inhibitors, and would have higher specificity in blocking the dual roles of EZH2. RNA-sequencing results also supported the notion of activation of AR targets via induction of EZH2 expression.

The ubiquitin–proteasome system (UPS) is the most prominent pathway for modulation of cellular EZH2 protein homeostasis. Ili-A decreased EZH2 protein levels in prostate cancer cells via accelerated degradation of EZH2. While MG132, a proteasome inhibitor, could reverse this effect. These results suggested that Ili-A could accelerate the degradation of EZH2 in prostate cancer cells through the proteasome pathway. We demonstrated that Ili-A suppressed expression of the EZH2 target genes *PLK1* and *AURKA* markedly, and inhibited the growth of PCa cells significantly via EZH2 degradation. Previous study demonstrated that EZH2 inhibitors such as EPZ and GSK126, though effective in blocking the enzymatic roles of EZH2, could not suppress EZH2-mediated activation of AR. Instead, they inadvertently increased AR expression (Ku et al., 2017). Our ChIP-PCR results indicated that EZH2 activates AR via its direct occupancy at the AR promoter, and promote an AR-dependent transcriptional network. Ili-A could repress EZH2 and other PRC2 components that methylate H3K27 and condense chromatin conformation. Inhibited expression of EZH2 decreased AR expression markedly at transcriptional and protein levels, and reduced expression of AR-activated genes, such as *KLK2* and *KLK3*.

Enzalutamide is a second-line AR antagonist used against CRPC. However, enzalutamide resistance is an urgent problem in treatment of PCa, which is induced mainly by AR point mutations, AR overexpression, and constitutively active AR splice variants (Visakorpi et al., 1995; Borgmann et al., 2018). Therapeutic strategies have focused on the combination of other reagents with enzalutamide for CRPC treatment (Han et al., 2017). In addition, several compounds have been shown to diminish drug resistance if added to enzalutamide treatment *in vitro* or *in vivo*, such as the CXCR7 inhibitor CCX771 (Luo et al., 2018), and AZD5363 (Toren et al., 2015), which targets the phosphoinositide 3-kinase/protein kinase B pathway. Studies have shown that EZH2 has a critical role in acquisition of enzalutamide-resistance in CRPC cells and drives enzalutamide-resistance (Varambally et al., 2002). Targeting EZH2 could overcome enzalutamide resistance in PCa cells. GSK126 combined with enzalutamide inhibits proliferation and colony formation of enzalutamide-resistant CRPC cells dramatically. In prostate cancer, EZH2 activates AR gene transcription through direct occupancy at its promoter.

Previous study demonstrated the effect of GSK126 and enzalutamide on disruption of EZH2 directly binds to androgen receptor (AR) in coordinating the activities of PRC2 complex and playing a central role in the interaction with H3K27 and enhancing H3K27me3 in CRPC cells (Liu et al., 2019); whereas silencing of EZH2 significantly decreased AR and the expression of downstream targets such as *PSA* and *TMPRSS2*. Furthermore, short-hairpin RNA-mediated depletion of EZH2 can enhance the efficacy of enzalutamide treatment in enzalutamide-resistant CRPC cells and xenograft tumors (Bai et al., 2019). Thus, beyond its transcriptional repressor function, EZH2 also acts as an activator for AR and its downstream targets facilitating growth of CRPC cells. Our studies demonstrate that combination treatment with Ili-A and enzalutamide dissociates the association between AR and members of the EZH2 downstream targets.

However, the EZH2 inhibitors-based treatment is basically ineffective against PCa, which limits its clinical application. Two EZH2 inhibitors, EPZ-6438 and GSK126, showed preliminary benefits in some hematological malignances with constitutive enzymatic activity of EZH2 (McCabe et al., 2012; Kurmasheva et al., 2017). Although the up-regulation of EZH2 is associated with advanced PCa and poor prognosis, the EZH2 inhibitors-based treatment is basically ineffective for PCa (Deb et al., 2013). Moreover, by employing 22Rv1 cell line-derived PCa xenograft models, we further revealed that Ili-A enhance the anticancer activity of enzalutamide in CRPC cancer models. This signifies that combined targeting of EZH2 and AR is an effective treatment option for CRPC.

## Conclusion

We demonstrated that Ili-A could be employed for CRPC therapy. Ili-A showed efficacious activity against PCa cells by abrogating EZH2/AR-mediated processes, and demonstrated a synergistic anti-PCa effect with enzalutamide *in vivo*. Taken together, these data suggest that Ili-A, a novel EZH2 inhibitor, could combine with enzalutamide and serve as a new therapeutic strategy for CRPC.

## Data Availability

The datasets presented in this study can be found in online repositories. The names of the repository/repositories and accession number(s) can be found below: NCBI (accession: GSE182064).
