# Supplementary material for: Ilicicolin A Exerts Antitumor Effect in Castration-Resistant Prostate Cancer Via Suppressing EZH2 Signaling Pathway
Source: Front Pharmacol. 2021 Oct 27;12:723729. doi: 10.3389/fphar.2021.723729 (PMC8578973; doi:10.3389/fphar.2021.723729)
Supplement: Supplementary file 1 [file DataSheet1.docx]

**Supplementary figure 1**

(A). Chemical activities in growth inhibition of C4-2B cells. (B). Cell viability were measured by Cell-Titer GLO (Promega) of C4-2B, 22Rv1 cells treated with the indicated concentrations of Ili-A(ASG3)for 4 days. The results are represented as means ±SD. **P* < 0.1, ***P* < 0.05, ****P* < 0.01 vs. control.


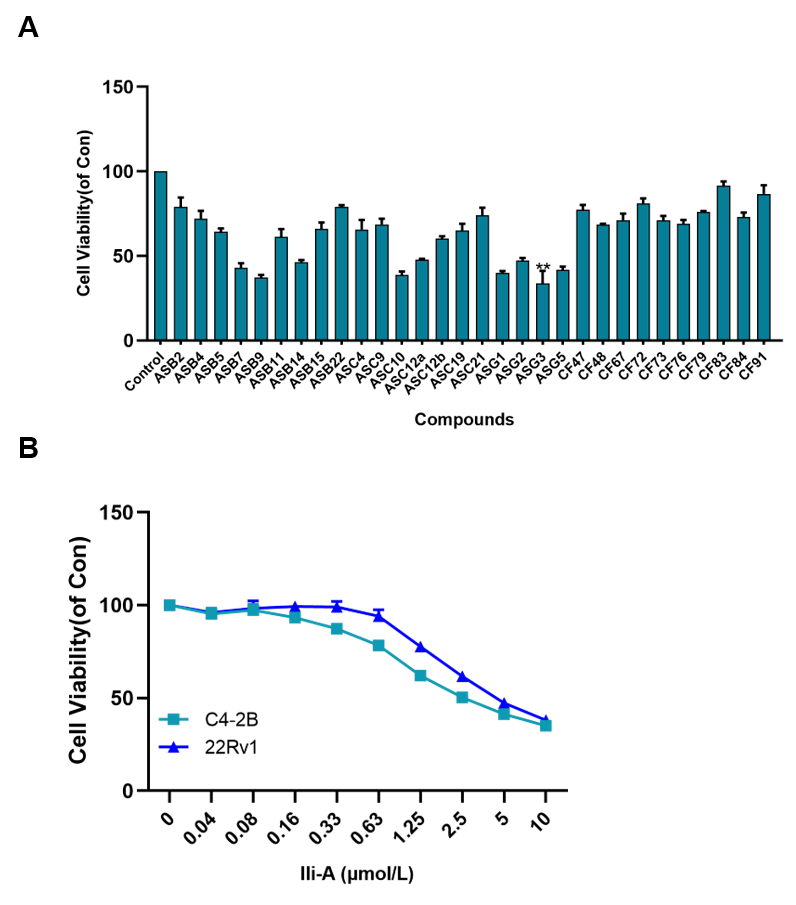


**Supplementary figure 2**.

Photographs of xenograft tissue harvested at day 22


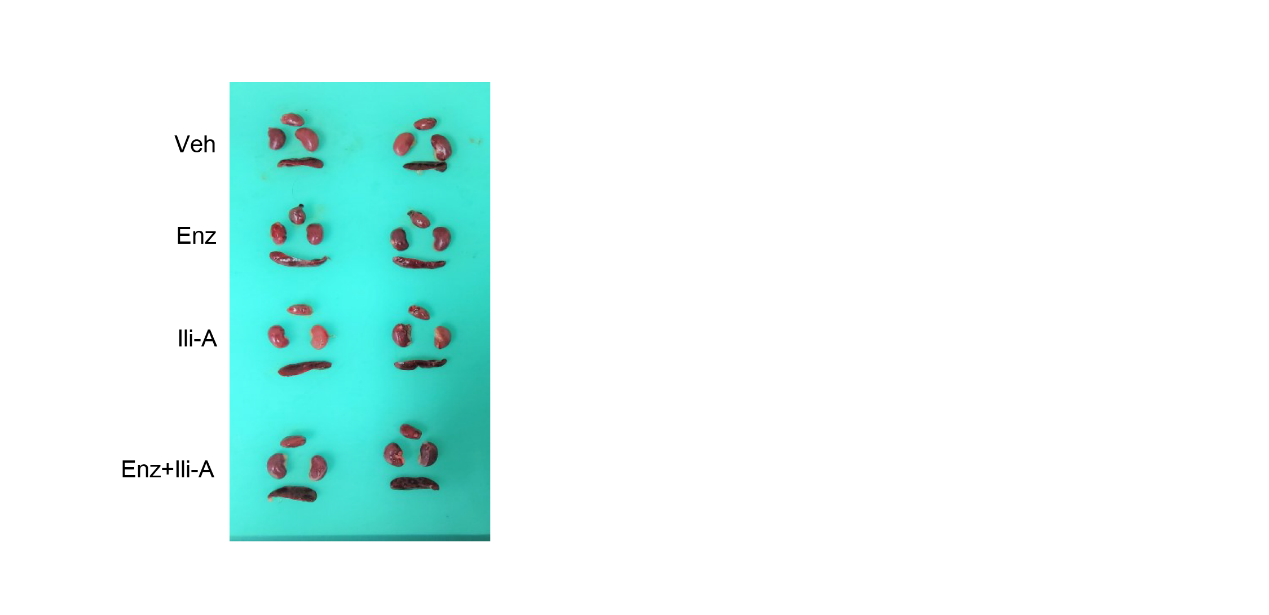


**Supplementary figure 3A**.

^1^H NMR spectrum of ilicicolin A (CDCl_3_, 700 MHz).

**Supplementary figure 3B**.

^13^C NMR spectrum of ilicicolin A (CDCl_3_, 175 MHz).

**Supplementary figure 3C**.

DEPT 135 NMR spectrum of ilicicolin A (CDCl_3_, 175 MHz).

**The physicochemical data of ilicicolin A**

Amorphous powder; ^1^H NMR (700 MHz, CDCl_3_): *δ*_H_ 12.68 (1H, s, 2-OH), 6.49 (1H, s, 4-OH), 2.58 (3H, s, H_3_-7), 10.12 (1H, s, H-8), 3.40 (2H, d, *J* = 7.0 Hz, H_2_-9), 5.06 (1H, t, *J* = 7.0 Hz, H-10), 5.06 (1H, t, *J* = 7.0 Hz, H-18), 1.99 (2H, m, H_2_-12), 2.07 (2H, m, H_2_-13), 5.22 (1H, t, *J* = 7.0 Hz, H-14), 1.99 (2H, m, H-16), 1.92 (2H, m, H-17), 5.08 (1H, t, *J* = 6.5 Hz, H-18), 1.58 (3H, s, H_3_-20), 1.66 (3H, s, H_3_-21), 1.56 (3H, s, H_3_-22), 1.79 (3H, s, H_3_-23); ^13^C NMR (175 MHz, CDCl_3_): *δ*_C_ 113.7 (qC, C-1), 162.3 (qC, C-2), 114.5 (qC, C-3), 156.5 (qC, C-4), 113.3 (qC, C-5), 137.7 (qC, C-6), 14.5 (CH_3_, C-7), 193.4 (CH, C-8), 22.1 (CH_2_, C-9), 120.8 (CH, C-10), 136.9 (qC, C-11), 39.8 (CH_2_, C-12), 26.8 (CH_2_, C-13), 124.4 (CH, C-14), 135.1 (qC, C-15), 39.7 (CH_2_, C-16), 26.5 (CH_2_, C-17), 124.0 (CH, C-18), 131.2 (qC, C-19), 17.7 (CH_3_, C-20), 25.8 (CH_3_, C-21), 16.1 (CH_3_, C-22), 16.3 (CH_3_, C-23).

**Supplemental table 1 Primers for ChIP assay and qPCR**

| **Primers for ChIP assay** | |
| --- | --- |
| AURKA chip -F | TTTGAATAAGCCAGAAACCA |
| AURKA chip -R | CTATTCTCCTGTCTCAGCCTCC |
| PLK1 chip -F | CCAGAGGGAGAAGATGTCCA |
| PLK1 chip -R | GTCGTTGTCCTCGAAAAAGC |
| AR chip -1.4KB F | CACAGGCTACCTGGTCCT |
| AR chip -1.4KB R | TCTGGGACGCAACCTCT |
| **Primers for qPCR** | |
| EZH2 qrt-F | GGACCACAGTGTTACCAGCAT |
| EZH2 qrt-R | TGAAAGTGCCATCCTGATCC |
| CCNB2 qRT-F | GGCTGGTACAAGTCCACTCC |
| CCNB2 qRT-R | CTTCTTCCGGGAAACTGGCT |
| FoxM1 qRT-F | TTCTCCTTTGCTTCCAGTTCA |
| FoxM1 qRT-R | CACTTTGATGGGTCTCGCTAA |
| CDKN1A qRT-F | ATG AAA TTC ACC CCC TTT CC |
| CDKN1A qRT-R | AGG TGA GGG GAC TCC AAA GT |
| PLK1 qRT-F | AAGAGATCCCGGAGGTCCTA |
| PLK1 qRT-R | GCTGCGGTGAATGGATATTT |
| AURKA qRT-F | TGGAATATGCACCACTTGGA |
| AURKA qRT-R | ACTGACCACCCAAAATCTGC |
| KLK3/PSA qRT-F | GGA AAT GAC CAG GCC AAG AC |
| KLK3/PSA qRT-R | CCA GCT TCT GCT CAG TGC TT |
| KLK2 qRT-F | CAACATCTGGAGGGGAAAGGG |
| KLK2 qRT-R | AGGCCAAGTGATGCCAGAAC |
| AR-FL-qRT-F | ACATCAAGGAACTCGATCGTATCATTGC |
| AR-FL-qRT-R | TTG GGC ACT TGC ACA GAG AT |
| β-Actin F | GAGAAAATCTGGCACCACACC |
| β-Actin R | ATACCCCTCGTAGATGGGCAC |

**Supplementary table 2 antibodies for immunoblotting**

| **Antibody** | **Vendor** | **Catalogue number** | **dilution** |
| --- | --- | --- | --- |
| AR | Cell signaling | #5153 | 1:1000 |
| cleaved-Caspase7 | Cell signaling | #9491 | 1:1000 |
| cleaved-PARP1 | Cell signaling | #9542 | 1:1000 |
| CyclinD1 | Santa Cruz | Sc-8396 | 1:500 |
| CyclinE2 | Santa Cruz | sc-9566 | 1:500 |
| GAPDH | Cell signaling | #2118 | 1:1000 |
| C-Myc | Santa Cruz | sc-40 | 1:500 |
| EZH2 | Cell signaling | # 5246 | 1:1000 |
| PLK1 | Santa Cruz | sc17783 | 1:500 |
| AURKA | Santa Cruz | sc-373856 | 1:500 |
| CCNB2 | Santa Cruz | sc-28303 | 1:500 |
| FOXM1 | Santa Cruz | sc-271746 | 1:500 |
| Histone H3K27 | Cell signaling | # 9733 | 1:1000 |
| Anti-mouse IgG | Cell signaling | #7076 | 1:5000 |
| Anti-rat IgG | Cell signaling | #7077 | 1:5000 |
| Anti-rabbit IgG | Cell signaling | #7074 | 1:5000 |
